# An Address before the Graduating Class of the Atlanta Medical College

**Published:** 1856-10

**Authors:** John L. Harris

**Affiliations:** Atlanta, Ga.


ATLANTA

Medical and Surgical Journal.

VOL. II.] OCTOBER, 1856. [NO. 2.

### ORIGINAL COMMUNICATIONS.

### ARTICLE I.

*An Address before the Graduating Class of the Atlanta Medical College.* By HON. JOHN L. HARRIS, of Atlanta, Ga.

*Gentlemen of the Graduating Class:*

I have been deputed by the Faculty to address you at this time. An hour of mingled congratulations and regret: congratulations, because, so far as your collegiate course is concerned, your duties have been accomplished, and your labors finished in a manner honorable to yourselves and highly gratifying to your professors; of regret, because the pleasant and interesting relation which has so long existed between you and them, rendered doubly pleasant by your docility, intelligence and gentlemanly deportment, this day determines.

And, gentlemen, permit me to add to this, the regrets of a community to which your singularly unexceptionable course has endeared you. You, this day, no longer aided by the wisdom and experience with which years and labor have endowed your professors, are called upon to answer to the roll call of your names on the stage of human action, and to take your position as actors in scenes which determine only with life. To us and to you this is a deeply interesting occasion. While we, tempered by age and experience, indulge a well founded and rational hope of your future success and usefulness, your souls are filled with the light of youthful and holy enthusiasm, that throws its roseate hue over coming events.

Beautiful, gloriously beautiful, is the high aspiration of a young heart, when with an eye of faith and hope fixed steadily on the star of honor, which gleams in the temple of Fame, with firm pace and brave soul, it addresses itself to the labor of the ascent.

Such noble impulses I read this day in the light of your eyes. You are full of hope. Standing, as you do, upon the threshold of practical life, it may not be deemed inappropriate to discuss some of the means by which future eminence may be obtained.

Human life is constituted and made up of the ordinary duties, obligations and incidents which, as a general rule, mark the existence of men and communities. Extraordinary events are, as the term imports, departures from the ordinary routine of life.

In forming your plans for the future, then, indulge in no wild dreams of critical convulsions and marvelous events, in which your talents shall blaze forth with meteoric ray. But calmly survey the field of enterprise that stretches itself out before your gaze, and promptly address yourself to each duty that may exact your attention. Labor and perseverence must underlie the structure designed for permanent usefulness. Solid and lasting materials must compose it.

The ice-built palaces of the Empress Catharine, the "imperial mistress of the fur clad Russ," were, as history reports, of exceeding magnificence and wondrous beauty; but they fell under the summer heat. Their materials were not enduring; they were as perishable in substance as they were glittering in aspect. *Omnia vincit labor*—a trite quotation, but the true index of the great Latin poet's success. Under the civil war which had desolated Rome, Virgil lost his meagre patrimony. Donations of lands to the soldiery, made the humble tenants of the soil homeless and houseless. Among the destitute and wretched, Virgil went to Rome to seek friends in that city to encourage and aid him. His poetic talents soon attracted the attention of his countrymen, and the first rude step was laid to be one in a series which carried him up the "steep where fame's proud temple shines afar." Untiring energy, and well directed labor acchieved for him a crown of fadeless laurel. He developed, illustrated and adorned the language of his native land. He wrote of flocks and herds, and charmed by the beauty of his language, and purity of his thoughts, the romantic youths of old Rome sighed for the shepherd's crook. He sung of arms, and a noble military enthusiasm was infused into the hearts of his countrymen. The little bee emblem of industry challenged his muse, and became poetic. He well said, "*In tenui labor, at tenuis non gloria.*" Labor is indeed and in truth the only certain mode of acquiring honorable distinction. And peculiarly so in what are known as the learned professions—such as medicine and law. The experience and wisdom of the great in each of these professions, expositions of abstruse and doubtful learning, analysis and comparisons ot existing facts to demonstrate unknown truths, present their ponderous volumes, and demand investigation. Here labor meets you, young gentlemen, and challenges application. You read and ponder upon what has been, that you may, by comparison, determine that which presents itself to you in doubtful aspect.

The collegiate course through which you have gone, is, indeed, but a formal introduction into your profession. The knowledge which you have here acquired, is but a mastery of elementary principles, by a skillful application of which, you can move on successfully and happily in the great field of science. He who believes that the possession of a diploma will constitute him a physician, or the having a license make him a lawyer, is sadly deceived. These are mere preliminaries to the battle of life—the buckling on of the armor, sword and spurs to the unfledged knight.

The future must demonstrate your right to the title in its broad and noble sense. One word here: be not deceived nor mislead by the influence of hope. You, in the discharge of your professional duties, must meet with casualties which will throw a temporary gloom over your feelings; if not, your lives will be miracles.

You must lose patients in your practice. Human wisdom and learning cannot avert divine decrees. When the race of life is run, mortal skill will be found unavailing to prolong its career. But, under all circumstances, in every instance, have the assurance of your minds and hearts, that you have done all "that does become a man; who dares do more is none."

Your convictions, honest convictions, of having done your whole duty, will support and sustain you even when casualties tread upon each other's heels in the practice of your profession. And, therefore, be not discouraged nor dismayed by accident, but press forward in the pathway of duty. The lawyer who never lost a case, and the physician who has never lost a patient, must, indeed, be of very limited practice.

Your duties are singularly interesting and impressive in their character, and your relations to your patients of the most intimate kind. The delicate machinery of this complex system is committed to your prudence and skill to correct its aberrations, and to repair its breaches. Here is to be exercised that knowledge which you have already acquired, and that which you shall hereafter acquire. The system is disarranged, disorganized. To apply the remedy, you must know the evil; to discover the existing cause of mischief, you must know well the condition of the several parts and members of the system when in a state of harmony and repose. Your collegiate course and accompanying investigatons and studies have imparted this necessary knowledge. It is for you, hereafter, to examine and determine each case submitted to you according to the rules and principles in which you have been instructed. And, thus, with necessary knowledge and a firm purpose, you will take your position in society.

The life of a physician is free from the turmoil and strife which marks that of the statesman or warrior. Yours is an humble but a happier and holier relation. You penetrate to the hearth-stones of your patients and friends, and are, in danger's hours, the rock under whose shadow they repose. A loved one is stricken down—a winged messenger flies to summon you to the scene of misery and woe: see with what intense agony the weeping watchers note the expression of your countenance as you examine your patient. You are now their judge. They await in voiceless suspense an intimation of your opinion. What is all this world to a mother, as she watches the convulsive twitches of her infant's limbs, and awaits your utterance in almost hopeless despair? These are hours and scenes that try men's souls. Every thing now rests with you; you feel the terrible responsibility which is upon you, and will, we doubt not, address yourselves like men to your tasks.

Again, you will, unless singularly fortunate, be called upon to expose your life to death, when it rides upon the breath of the pestilence. Others may seek safety in flight; the mother may desert her offspring; the husband fly from his wife, as was done in the streets of London, in the days of the plague; but you, young gentlemen, must face the storm of death. Your persons must be exposed to every hazard. Your cheeks may grow pale, but your hearts must be firm. The path of duty is plain before you, and your profession enjoins on you to pursue it. You must be martyrs for humanity, if God so wills it. Such is the high responsibility of your noble profession. How different the scenes under which you are called upon to meet not only death, but loathsome and lingering disease, from those which surround the soldier as he faces his foe.

On the battle field we have the "spirit stirring drum, the ear piercing fife," the bugle's martial blast, waving banners, and all the pomp and circumstance of war which would make even the timid take heart of grace, and plunge into the fight. But reverse the picture; see the physician, wearied and worn under the protracted labors incident to the prevalence of a scourge like yellow fever or cholera. He staggers home to repose his sinking frame, and refresh his wearied limbs. There stands a messenger, with faltering tongue and palid lip, he whispers, come, come! Almost exhausted, the physician would fain excuse himself. "An hour's respite and he will go." The messenger, like the importunate widow, will take no denial. With trembling finger he points to the habitation of squalid poverty and want, where death holds his grim carnival. Those pallid lips once more unclose, "My wife and children are stricken, come." Gold cannot buy what charity gives. The physician follows him to the scene of woe—God shield the poor pilgrim on thy perilous errand—they arrive at the threshhold. Hush! on stealthy toe, and with cat-like tread, see him as he creeps firmly into the very jaws of death; the choked atmosphere is charged with disease. See him as he bends over his patient, administer what skill prescribes—encourages with words of hope, and once more, fainting and exhausted, emerges into the open air.

This, young gentlemen, is courage—the highest of all courage—moral courage—the firmness of Latimer and Redly among the flames. Here is the high, noble self-sacrifice which duty may call on you to offer. Here is whence the light emanate, which sheds its halo around the true physician's head. It is among such scenes as these, when plagues visit our people, that we expect noble acchievements by you in the cause of medical science and humanity. Hence, this is a deeply interesting occasion to us and to you.

Again, we shall watch your course with deep anxiety, being the graduates of a young college, now taking proud position with her sister colleges. We have the fullest confidence in you. We are glad that the present character of our college is committed to such worthy hands. Not one shadow dims the brightness of our hope for you, and, through you, this young institution. You will be pointed at, and known as graduates of the Atlanta Medical College; *your* destiny is *hers; she* is willing to risk her character in *your hands.* Will you accept the trust? You will meet moustached gentry fresh from Philadelphia or Paris, who may sneer at the graduate of an up-country college. I pray you be not troubled; the first rude blast which passes will sweep away these gossamers: they have mistaken their calling; they may make fancy doctors for a ball room, to pour balm of a thousand flowers upon some fainting lady's lips. The true physician is made of sterner stuff. To be a physician, it is necessary to be a *man:* I use the term in its broadest, noblest sense. We rejoice, young gentlemen, that you have chosen to complete your course, and graduate at your college.

The establishment of a college here was, to some extent, a work of necessity, and certainly of enlightened patriotism, occupying a central position, as Atlanta does in some degree, to the whole State, and certainly to more than half its voters. It was right and proper, that the up-country of Georgia should not longer be subordinated to the interests of the South and East. But to avoid all misconstruction, to shun even the appearance of a collision in interest, or a want of harmony in action at home, it was thought proper to establish a course of summer lectures, and, by this means, supply a desideratum long wanted in the South. Southern gentlemen may now, upon a Southern soil, in a Southern college, attend a course of summer lectures. Do not misconstrue me. I have no hostility to Northern institutions; but I do depreciate that spirit of servile dependence which has so long hung like an incubus upon the South, draining her resources, and dishonoring her character. It was a want universally felt and acknowledged. The establishment of this college was intended to supply this deficiency in the South. Its past and present position is flattering indeed; and we feel that we might safely predict for it a career of unprecedented glory and success. It ought to be, and must be, from its peculiar character, the recipient of unexampled patronage. Southern men, every where, alive to the true interests of our people, will encourage and adopt this institution as the foster child of the South. They have watched with painful solicitude, the faint pulsations of the new born infant. They see that there is life; its limbs are acquiring strength and activity, and it is perfect in all, save the length of its arms. In this particular, it is truly a little monster; for its arms will reach from the wave-lashed shore of the Chesapeake to the everglades of Florida. Truly, men did prophecy evil things concerning it, but they, who with mocking tongue and sneering lip, predicted evil, declaring it would prove a failure, an abortion, furnish but one more striking illustration of the prophet's truth: "Their young men shall see visions, and their old men dream dreams." It is of strong and sturdy growth, and rising in power, has dismayed and crushed out its enemies, even as the infant Hercules strangled the serpent. Its course is upward and onward—going on conquering and to conquer. It has taken root in the heart of the people. Its friends are multiplying every where, and every thing indicates a glorious future.

You, young gentlemen, are graduates of this institution; she is proud of her sons. Like the Spartan mother, she gives you your arms to-day, with the injunction, "return with them, or upon them." You are armed for the conflict of earth; the world lies before you. Henceforth, you are the architects of your own fate.

> "In the world's broad field of battle,
>   In the bivouac of life,
> Be not like dumb driven cattle,
>   But be Heroes in the strife.
> Trust no future, however pleasant,
>   Let the dead past bury the dead!
> Act—act in the living present,
>   Heart within, and God o'er head.

